# MAJOR STUDY FINDINGS ON THE ASSOCIATION BETWEEN MEDICAID PAYMENTS AND COSTS

**DOI:** 10.1093/geroni/igae098.2004

**Published:** 2024-12-31

**Authors:** Edward Miller, Marc Cohen, Elizabeth Simpson, Sara Karon, Iara Oliveira, Judith Dey, Marie Squillace, John Bowblis

**Affiliations:** University of Massachusetts Boston, Boston, Massachusetts, United States; University of Massachusetts Boston, Boston, Massachusetts, United States; University of Massachusetts Boston, Boston, Massachusetts, United States; RTI International, Research Triangle Park, North Carolina, United States; Office of Behavioral Health, Disability, and Aging Policy (BHDAP), Washington, District of Columbia, United States; US Department of Health and Human Services, Washington, District of Columbia, United States; Office of the Assistant Secretary for Planning and Evaluation, Washington, District of Columbia, United States; Miami University, Oxford, Ohio, United States

## Abstract

In 2019, Medicaid payment rates covered about 82 cents per dollar of estimated nursing home (NH) reported Medicaid costs. Roughly 40% of NHs had Medicaid payments covering 80% or less of their reported costs; 51% of NHs had 80-100% of their reported Medicaid costs covered; 8% of NHs had payments exceeding reported costs. Not-for-profit NHs had the lowest Medicaid payment-to-cost ratio (.76) compared to for-profit (.83) and government-owned (.80) NHs. NHs with the highest Medicaid payer mix (80-100%) had a higher Medicaid payment-to-cost ratio (.86) than NHs with the lowest Medicaid payer-mixes below 50% (.75). NHs with total nursing staff levels below 3.00 hours per resident day had the highest average Medicaid payment-to-cost ratio (0.85); NHs with nursing staff levels above 4.0 hours had the lowest (0.77). NHs with the lowest (1-star) quality ratings had the highest average Medicaid payment-to-cost ratio (0.83); NHs with 5-star ratings had the lowest (0.80).

